# Urban health in Africa: a critical global public health priority

**DOI:** 10.1186/s12889-019-6674-8

**Published:** 2019-03-25

**Authors:** Jo Vearey, Isaac Luginaah, Ng’weina Francis Magitta, Dativa J. Shilla, Tolu Oni

**Affiliations:** 1 African Centre for Migration & Society, University of the Witwatersrand, Johannesburg, South Africa; 2 Department of Geography, The University of Western Ontario, London, Ontario Canada; 3 Department of Biochemistry, Mbeya College of Health and Allied Sciences, University of Dar es Salaam, Mbeya, Tanzania; 4 Department of Environmental Chemistry, Dar es Salaam University College of Education, Dar es Salaam, Tanzania; 5 School of Public Health and Family Medicine, University of Cape Town, Faculty of Health Sciences, Room 4.41, Entrance 5, Falmouth Building, Cape Town, South Africa; 6 Stellenbosch Institute for Advanced Study, Stellenbosch, South Africa; 7 MRC Epidemiology Unit, University of Cambridge, Cambridge, UK

**Keywords:** Urban health, Africa, Sustainable development goals, Universal health coverage, Agenda 2063, African union, New urban agenda, Social determinants of urban health

## Abstract

The African continent is predicted to be home to over half of the expected global population growth between 2015 and 2050, highlighting the importance of addressing population health in Africa for improving public health globally. By 2050, nearly 60% of the population of the continent is expected to be living in urban areas and 35–40% of children and adolescents globally are projected to be living in Africa. Urgent attention is therefore required to respond to this population growth - particularly in the context of an increasingly urban and young population. To this end, the Research Initiative for Cities Health and Equity in Africa (RICHE Africa) Network aims to support the development of evidence to inform policy and programming to improve urban health across the continent. This paper highlights the importance of action in the African continent for achieving global public health targets. Specifically, we argue that a focus on urban health in Africa is urgently required in order to support progress on the Sustainable Development Goals (SDGs) and other global and regional public health targets, including Universal Health Coverage (UHC), the new Urban Agenda, and the African Union’s Agenda 2063. Action on urban public health in Africa is critical for achieving global public health targets. Four key research and training priorities for improving urban health in Africa, are outlined: (1) increase intersectoral urban health literacy; (2) apply a healthy urban governance and systems approach; (3) develop a participatory and collaborative urban health planning process; and, (4) produce a new generation of urban health scholars and practitioners. We argue that acting on key priorities in urban health is critical for improving health for all and ensuring that we ‘leave no-one behind’ when working to achieve these regional and global agendas to improve health and wellbeing.

The Research Initiative for Cities Health and Equity in Africa (RICHE Africa) Network aims to support improvements in urban health across the continent. The RICHE Africa Network is made up of both early career and established researchers working to support evidence-informed urban public health policy and planning processes in Africa. In this brief paper, we argue that a focus on urban health in Africa is required to support progress on the Sustainable Development Goals (SDGs) [1] and other global health targets, including efforts to achieve Universal Health Coverage (UHC) [2] the Habitat III New Urban Agenda (NUA) [3] and the African Union’s Agenda 2063 development plan [4]. We outline four key research and training priorities for improving urban health in Africa, that will ensure we ‘leave no-one behind’ when working to achieve these goals.

## The importance of Africa for global public health

Between 2015 and 2050, over half of the expected global population growth will be in Africa [5], highlighting the importance of addressing population health on this continent for improving global public health. This increase in population growth in Africa is characterised by two distinct features. Firstly, while Africa is currently the least urbanised region of the world, it represents the fastest urbanising continent, with 56% of the population of the African continent projected to be living in urban areas by 2050 [6]. Secondly, this population growth is characterised by a demographic youth bulge with 35–40% of children and adolescents globally projected to be living in Africa by 2050 [7]. These features represent an opportunity for improving health in the urban setting. Urbanisation can result in an “urban advantage” with improved access to healthcare services, education and employment opportunities, and strengthened social connections (compared to their rural counterparts), while the youth demographic dividend represents an opportunity for economic growth through a growing skilled workforce. However, this potential is as yet unharnessed with increasing intra-urban inequities - evidenced by the high proportion of African urban residents, including those migrating into cities, living under conditions of informality, such as unsafe human settlements, and poor access to services that increase vulnerability to poor physical, mental and social health - resulting in an “urban penalty” [8]. This penalty is further exacerbated by high rates of unemployment and high-risk health behaviours among young people. This picture has become increasingly complex in the African context, with the rise of chronic non-communicable diseases (NCDs) – such as diabetes, hypertension and heart disease - affecting both non-poor and poor alike [9] and a rising prevalence of infectious/NCD multimorbidity in increasingly younger age groups [10].

## A focus on urban health in Africa

Cities are, therefore, critical spaces for action to achieve the SDGs, UHC, and NUA in Africa and, as a result, globally: both in terms of the numbers and demographics of people living in urban areas now and in the future, and as a result of increasing levels of urban health inequities. Furthermore, the first goal set out by the African Union in the Agenda 2063 development plan [4] highlight the need to focus on inclusive growth and sustainable development, through ensuring a high standard of living, and quality of life, sound health and well-being. This aspiration recognizes cities as important hubs of cultural and economic activities, but also as sources of exposures that determine health such as the nature of the food system and exposure to high sugar and salt foods; the quality of housing and contribution to social cohesion, contributors to stress and exposure to noise, air, and water pollution [11].

The RICHE Africa Network is working to develop a common research and training agenda that aims to generate empiric evidence that presents options for improving the health and wellbeing of urban residents across the continent. Emerging out of a meeting of the International Council of Science’s Urban Health and Wellbeing scientific committee and a Future Earth Knowledge Network meeting on Urban Health in Xiamen, China in December 2017, the RICHE Africa Network has identified four key research and training priorities for improving urban health towards achieving the SDGs in Africa [1]: increase intersectoral urban health literacy [2]; apply a healthy urban governance and systems approach [3]; develop a participatory and collaborative urban health planning process; and [4], produce a new generation of urban health scholars and practitioners.

## Urban health priorities to achieve global and regional sustainable development goals in Africa

### Increase intersectoral urban health literacy

To address urban health in Africa we need to improve urban health literacy across multiple sectors and at multiple levels, inclusive of public, private and civil society actors. Urban health is more than meeting the health needs of people living in cities. It is, in itself, an approach that recognises the physical, social and economic urban environments as determinants of health and of health equity. Driven by a public health and social justice agenda, an urban health approach recognises that cities are inherently unjust spaces typified by inequality and inequity, and the resultant intra-urban disparities in health. The complexity of African cities needs to be understood, by citizens and policymakers alike, from a Social Determinants of Urban Health (SDUH) [12] perspective, that will allow for investigation and identification of the interlinked multi-level and multi-sectoral determinants of urban health (exposures) and the pathways that connect them to health outcomes (systems), with the objective of improving equity in urban health [8, 10]. Achieving this objective will require partnerships with sectors and organisations across public policy, private, and civil society actors, who are well positioned to “move the needle” on these issues. There is therefore a need for research into the knowledge and attitudes of key stakeholders across these sectors, in order to inform the development of appropriate strategies to improve health and SDUH literacy. Such interventions should target children and youth in the general population in order to bolster prevention efforts at the individual, community, and structural levels, and to empower communities to apply an SDUH and rights-based lens to better advocate for improved living and working conditions in the city.

### Apply a healthy urban governance and systems approach

The rapid growth rate of many African cities results in existing urban governance systems struggling to meet demand, with reactive - rather than proactive - city planning being implemented. This governance approach does not adequately consider the SDUH and health outcome implications of interventions and results in further deepening experiences of deprivation and inequity. These deprivations affect the ability of some residents – particularly those residing on the physical and social periphery of the city - to thrive [13]. An SDUH lens facilitates the identification and analysis of the multiple factors, such as diet, physical activity, natural and built environments affecting equity in health, and calls for a multilevel approach - beyond the individual - to develop interventions to address these foundational health determinants. This includes addressing commercial determinants of health, and developing improved public policy that is aligned for health and sustainable development. Key to this is the role of governance – “the institutions and processes through which societies manage the course of events” [14]. Governance is about the ways in which multiple actors work together: the state; civil society; academia; the private sector; and, community-based organisations. In the context of cities, evidence indicates that improved urban governance is required to inform sustainable and healthy urban planning [14]. Such healthy urban governance requires improved understanding of city dynamics – urban growth, migration and mobility, resource mobilisation and use, informality, ageing populations - and engagement with social, physical, environmental, and economic security, all of which are key, interlinked determinants of urban health.

Unpacking and understanding the complexity of health in African cities, and the ways in which governance structures affect equity in health, requires research to explore and understand the relationships between exposure(s) and the distribution(s) of health outcome(s) as well as the inter-relationships between city, provincial, and national governments. Health inequities in the city are the outcome of multiple system failures, requiring a systems approach to research and action [8, 15]. Critical to this is the availability of quality integrated, intersectoral and longitudinal data that can be disaggregated and used for the exploration of multi-causality pathways. Such evidence can be used to inform policy development and prioritisation, as well as to evaluate the health (equity) impact of intersectoral urban interventions.

### Develop a participatory and collaborative urban health planning process

We need to move beyond existing, sectoral approaches to health in urban settings and develop contextually relevant city health plans. Key here is the need for multiple and collective actions to improve global health; action on Goal 3 – *to ensure healthy lives and promote wellbeing for all at all ages* - has been identified as central to achieving progress across all SDGs [16]. This requires a focus on the ways that local government interfaces with the city as a service provider, and the modes of city planning and governance. Local-level researchers, practitioners and city managers should be involved in the collective and participatory development of urban health plans [17] to support a systems approach for engagement and action between sectors [9, 15]. Such plans should include the identification of short, medium, and long-term goals, with a clear guiding framework for deliverable health outcomes. Ideally, these plans and the associated activities and outcomes should be integrated into existing performance management systems at the local government level across health and non-health sectors. Participatory approaches should crucially involve the public; this recognises the potential to mobilise young people to act both as citizen scientists - applying innovative approaches to collection of SDUH data in the city - and as engaged members of their community who can provide informed input into proposed urban health planning processes.

### Produce a new generation of urban health scholars and practitioners

We need to develop improved undergraduate and postgraduate training in order to produce a new generation of African urban health scholars and practitioners who are literate across sectors and disciplines, and have specialised skills in systems thinking in order to conduct implementation and transdisciplinary research. Critically, these scholars and practitioners need to be able to act as interlocutors – catalysts – between diverse urban governance actors, and be able to meaningfully lead engagement with different stakeholders on why urban health matters, and why often challenging cross-sectoral collaboration should be made a priority in their respective mandates.

Moving forward, we call on the global health community, and especially African governments to consider the African urban health agenda. In order to make any gains on global and regional sustainable development and health agenda – including the SDGs, UHC, and Agenda 2063, systems approaches to improving urban health and addressing urban justice in Africa should be prioritized.

## Abbreviations

NCDs: Non-communicable diseases
RICHE: Research Initiative for Cities Health and Equity
SDGs: Sustainable Development Goals
SDUH: Social Determinants of Urban Health
UHC: Universal Health Coverage
